# Prediction of Oil Palm Yield Using Machine Learning in the Perspective of Fluctuating Weather and Soil Moisture Conditions: Evaluation of a Generic Workflow

**DOI:** 10.3390/plants11131697

**Published:** 2022-06-27

**Authors:** Nuzhat Khan, Mohamad Anuar Kamaruddin, Usman Ullah Sheikh, Mohd Hafiz Zawawi, Yusri Yusup, Muhammed Paend Bakht, Norazian Mohamed Noor

**Affiliations:** 1 School of Industrial Technology, Universiti Sains Malaysia, Gelugor 11800, Malaysia; nuzhat_khan@student.usm.my (N.K.); yusriy@usm.my (Y.Y.); 2 School of Electrical Engineering, Universiti Teknologi Malaysia, Johor Bahru 81310, Malaysia; muhammad.paend@buitms.edu.pk; 3 Department of Civil Engineering, Universiti Tenaga Nasional, Kajang 43000, Malaysia; 4 Faculty of Information and Communication Technology, BUITEMS, Quetta 87300, Pakistan; 5 Sustainable Environment Research Group (SERG), Centre of Excellence Geopolymer and Green Technology (CEGeoGTech), Faculty of Civil Engineering Technology, Universiti Malaysia Perlis, Arau 01000, Malaysia; norazian@unimap.edu.my

**Keywords:** oil palm, crop yield, prediction, machine learning, precision agriculture, sustainability

## Abstract

Current development in precision agriculture has underscored the role of machine learning in crop yield prediction. Machine learning algorithms are capable of learning linear and nonlinear patterns in complex agro-meteorological data. However, the application of machine learning methods for predictive analysis is lacking in the oil palm industry. This work evaluated a supervised machine learning approach to develop an explainable and reusable oil palm yield prediction workflow. The input data included 12 weather and three soil moisture parameters along with 420 months of actual yield records of the study site. Multisource data and conventional machine learning techniques were coupled with an automated model selection process. The performance of two top regression models, namely Extra Tree and AdaBoost was evaluated using six statistical evaluation metrics. The prediction was followed by data preprocessing and feature selection. Selected regression models were compared with Random Forest, Gradient Boosting, Decision Tree, and other non-tree algorithms to prove the R^2^ driven performance superiority of tree-based ensemble models. In addition, the learning process of the models was examined using model-based feature importance, learning curve, validation curve, residual analysis, and prediction error. Results indicated that rainfall frequency, root-zone soil moisture, and temperature could make a significant impact on oil palm yield. Most influential features that contributed to the prediction process are rainfall, cloud amount, number of rain days, wind speed, and root zone soil wetness. It is concluded that the means of machine learning have great potential for the application to predict oil palm yield using weather and soil moisture data.

## 1. Introduction

Crop yield prediction [1,2] is a critical yet fascinating issue due to its requirement for long-term intensification and optimal use of natural resources [3]. Many stakeholders in the agri-food chain, including agronomists, farmers, product exporters, and policymakers, benefit from crop yield forecasts [4,5]. Various crop-specific characteristics, environmental conditions, and management practices influencing crop production [6,7] are some of the confounding factors for developing a prediction model [8]. Recent research highlighted the need for weather-based crop yield forecasting as one of the ways to minimize the negative effects of climate variability and extremes under current climate conditions [7,9,10]. At the same time, yield forecasting [9,10] is emphasized as an adaptation technology to climate change for global food security [11,12]. The approaches to anticipate crop yield include: (1) field surveys [13], (2) crop growth models [14], (3) remote sensing [15], (4) statistical models, as well as (5) the combinations of these approaches [16,17,18,19,20,21]. For instance, field surveys are used to observe the ground truth with human expertise [5]. Meanwhile, the crop growth simulation models are governed by the environment, management strategies, and agronomic principles [22]. On the other hand, remote sensing techniques capture the current status of crops to estimate the final yield [23,24]. One of the main limitations of the aforementioned methods is regarding their incompetence to capture fluctuating abiotic environmental factors [25]. Recent advancements in big data and machine learning have introduced precision agriculture [9], wherein machine learning models are applied for crop yield prediction [26]. Machine learning combines the strengths of the previous methods, such as remote sensing and growth simulation models, with data-driven modeling to produce reliable forecasts [27,28,29]. Machine learning algorithms use outputs of conventional methods as features and try to approximate a function that connects predictors (features) to the target (crop yield) [30,31]. Numerous machine learning and deep learning models have been proposed for environment-based yield prediction of various crops [32,33,34]. However machine learning is underutilized for predictive analysis of oil palm [35]. Despite all technology gaps, the oil palm industry is growing rapidly to fulfill the increasing global demand. Conversely, this crop is threatening tropical forests, biodiversity, and associated ecosystems [36]. One of the major challenges related to oil palm crop is its unimpeded expansion [35] which has violated a perceived moral obligation of sustainability [37,38]. Therefore, the oil palm sector is under increasing environmental, economic, and political pressures for endangering the ecological future [39]. The long-term viability and resiliency of the oil palm industry is determined by the capability of estate managers to make strategic decision and procedural changes [40,41]. In this regard, the most suitable solution rather than opening new lands, is acclimating the latest technology to elevate the yield by reducing the gap between actual yield and potential [42,43,44]. However, some factors, including fluctuating weather, may influence the outcomes significantly [45]. Therefore, data-intensive frameworks which are created in the context of the agro-environmental domain for weather-based oil palm yield forecasting are required. Then, evidence-based decision-making can be achieved by associating machine learning with real data [46]. So far, limited research had been conducted for oil palm yield prediction using a Bayesian network and artificial neural network (ANN) [47,48]. Similarly, OettliBehera, and Yamagata explored yield trends statistically by involving climate change to predict country-level oil palm yield [49]. Existing statistical models uncovered linear patterns, but failed to interpret nonlinear dependencies in the data [50,51]. Data greedy ANN, on the other hand, is unexplainable and unaccountable owing to the “black box” effect [52]. To deal with the shortcomings of existing models, a spatially transitional machine learning model integrated with automated machine learning (auto-ML) method [53] is presented in this paper. The methodology is proposed to develop a robust yield forecasting model according to the meteorological variability of the site. In this research, we comply with the need for a modular prediction workflow that can be used to: (1) better understand the convenience of multisource data, (2) improve data quality through a set of preprocessing techniques, (3) select significant feature subset, (4) select appropriate machine learning model by comparing several suitable models automatically, and (5) predict oil palm yield using historical observations. A conventional machine learning regression approach was combined with auto-ML to establish a precise yet flexible prediction model designed for oil palm fresh fruit bunch (FFB) yield. To the best knowledge of authors, the proposed framework has not been reported for crop yield prediction before. In this work we have addressed the following existing problems as indicated by previous research: (1) data scarcity, (2) machine learning application for predictive modeling of oil palm, and (3) designing a generic workflow in pursuance of reusability. In addition, the applicability of machine learning algorithms in predicting the oil palm yield from real data was evaluated with primary data.

In addition to the predictive modeling of oil palm data, we developed several machine learning and ensemble models and compared their performance for oil palm yield prediction. The major objectives were to: (1) develop a hybrid approach to forecast oil palm yield from actual data using machine learning techniques at the state level, (2) identify the suitable prediction models with reasonable explainability, (3) quantify the relation between meteorological predictors and the yield variability, and (4) apply the feature selection to detect significant predictors. It is verified that weather parameters can be used as a predictive measure for oil palm yield. The results from this study will contribute towards a better understanding on the relationships between oil palm yield and environmental factors.

Crop agronomic management, such as planting density, fertilizer application, and irrigation can be used to offset the loss in yield due to the weather effects. The implication of a generic machine learning workflow for oil palm yield prediction will provide the foundation for flexible crop yield forecasting. The proposed novel approach can assist policymakers for: (1) field management, (2) minimizing the negative effects of weather, (3) timely crop handling including fruit harvesting, storage, processing, and transportation management, and (4) import/export.

The manuscript is organized as follows: following the “Introduction” in the Section 1, while the “Materials and methods” are explained in Section 2. The Section 3 is devoted to “Results”, and the “Discussion” is presented in Section 4, followed by the “Conclusion” in Section 5.

## 2. Materials and Methods

First, we investigated the agronomic principles of crop modelling to identify features that are particularly useful for machine learning. Second, a flexible configurable design allowed selection of optimum feature subset. We developed two machine learning models for predicting the oil palm yield in Pahang, Malaysia by running different experiments. Next, the models were evaluated based on the multiple evaluation metrics and then compared with other similar models for true validation of the learning process. In addition, the performance of the models was compared with different state-of-the-art regressors. The effects of the numerous elements on prediction accuracy were revealed after a rigorous statistical and technical evaluation of input features and model training process. 

### 2.1. Study Site

**Figure 1:**
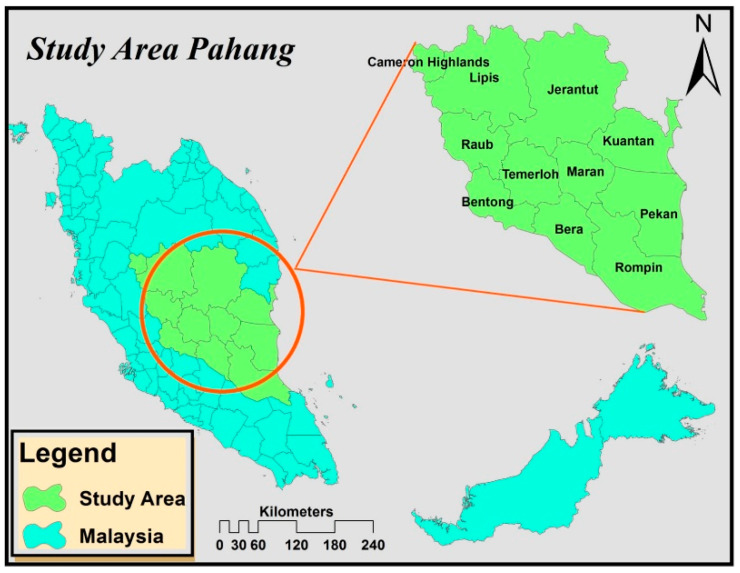
Study area.

Pahang is a state with a total area of 35,965 km^2^ and located at 4°11′10′′ N and 104°03′45′′ E on the east coast of peninsular Malaysia [54,55]. The two most important land uses in Pahang are forest and palm oil, both of which contributed to the food and the state’s revenue. At the same time, the synergy between oil palm and forest, together with climate change, is complicating the implementation of policy reforms in Pahang. The state includes 74 forest reserves with 10 virgin forests, the largest of which is Taman Negara Pahang, a part of the Central Forest Spine blueprint [56]. Despite all efforts, forest conservation in Pahang remains ineffective, with oil palm development serving as one of the principal causes of deforestation, emitting 110.6 million Mg CO_2_ across the Malaysian Peninsula between 2005 and 2015 [57,58]. Out of Malaysia’s 5.87 million hectares of oil palm, 15% are planted in Pahang, which accounts for 23.4% of the state’s GDP [59,60,61]. Despite this, Pahang’s palm oil sector is jeopardized by stagnating crop production on the account of climate change. This is the motivation to select Pahang as the study area (Figure 1).

### 2.2. Multi-Source Datasets

Multisource historical data for this research was obtained from the Malaysian Palm oil Board (MPOB), Meteorological department Malaysia (MET) sourcing three weather stations, and NASA Data Access Viewer (agroclimatology). The data were comprised historical observations (for a period of 35 years) including monthly FFB yield (tons/hectare) records and monthly average values, consisting of 13 weather-related parameters i.e., specific humidity, relative humidity, precipitation, surface pressure, temperature range, minimum temperature, maximum temperature, earth skin temperature, radiative flux (solar radiation), rainfall, wind speed, number of rainy days, and cloud amount. From the soil data, the three soil moisture related features include surface soil wetness, profile soil moisture, and root zone soil wetness, while one time-related feature is the date in the range of 01/1986 to 12/2020. All numerical features contained discrete values, except for the index column which is in date-time format. A detailed summary of input data is presented in Table 1. 

### 2.3. Prediction Framework

**Figure 2:**
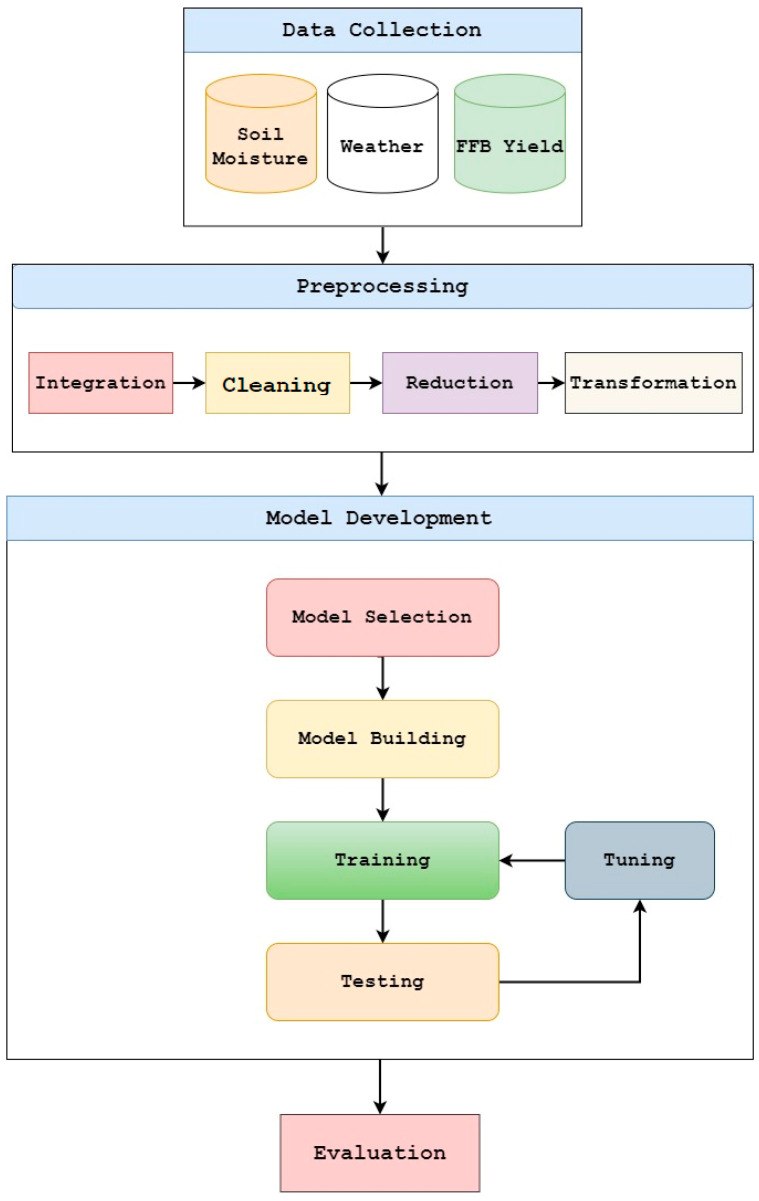
Proposed workflow.

The overall framework is broadly categorized into two major steps: (1) data preprocessing and (2) model development. A detailed description of the main steps and their sub-steps is presented in Figure 2 and explained in the subsequent section.

### 2.4. Data Pre-Processing

The fundamental aim of data preprocessing was to transform the raw data into well-structured meaningful information. Initially, the raw data was contained within 420 training points and 18 columns, including the target variable. The raw data was mainly preprocessed in four major steps that are described in the subsequent sections.

#### 2.4.1. Integration

Data from multiple sources were combined into a single database after schema integration and unit conversion. Several problems to be considered during data integration were inconsistent temporal resolution, measuring units, entity identification, detecting, as well as resolving data value and type. For example, the date format in different databases needs to be unified. Similarly, sources contained surface pressure measured in two different units. Therefore, the hectopascal (hPa) required unit conversion into the kilopascal (kPa).

#### 2.4.2. Data Cleaning

Data cleaning is a key step before implementing machine learning. In this process, data was prepared for prediction by removing the data points far beyond the normal range, commonly known as outliers. This approach is often used to eliminate data points that are inconsistent with other members in the same data set. The existence of outliers in the data degrades machine learning predictions, potentially leading to incorrect conclusions [62]. For real-world data, extreme weather conditions were identified as outliers [63] For this reason, significant outliers in data features were removed using the Z score method [64]. Although outliers removal reduces data size, it could, also, improve the data quality.

#### 2.4.3. Data Reduction

Weather and soil moisture data are represented by numerous variables that are not equally important in yield prediction. Furthermore, for small sample sets, machine learning algorithms commonly underperform upon the existence of redundant or less explanatory features. Thus, it is of supreme importance to find important features and discard redundant ones that might decrease the prediction accuracy [65]. The effective technique to reduce feature dimension is by discarding features that are not strongly related to the target, or carry similar information as other stronger features [66]. The Boruta algorithm [67] was applied to select an optimum feature subset. Boruta algorithm is a wrapper around random forest algorithm that works well for classification and regression problems. In data reduction, undesirable features were removed iteratively. This process returned compact data to make prediction easier.

#### 2.4.4. Data Transformation

In the process of data transformation, the structure/format of the data was changed. This step was needed to ensure equal distribution of data values. The diversity in feature values could cause bias during the model training process. For instance, in data rainfall was measured in millimeters and its value ranged from 3.36 (in drought) to 997 (abundant rain), depending on weather conditions. On the other hand, values of soil moisture remained in the range of 0.56 and 0.99 which are very low compared to rainfall feature values. In this case, high feature values were given more weightage than low feature values by the models. To overcome this issue, feature normalization was performed using the Min–Max scaling method that transformed each feature value within a common scale to the range of 0 and 1 using the following mathematical formula:

(1) $$X=\frac{x-x_{min}\;}{x_{max}-x_{min}}$$

where *X* is the new normalized data, *x* is the range of original data, while *x_min_* and *x_max_* are the lowest and highest values of the features, respectively [68]. Moreover, scaled data was randomly divided into two sets, of which 70% data was utilized to train the models using repeated *k*-fold cross-validation technique [69] with 10 folds. The 10-fold cross-validation is a technique for evaluating machine learning models of a small sample of data. In this process, training data was divided into 10 groups to train the machine learning models. In addition, the remaining 30% of the test data was considered to verify prediction accuracy.

### 2.5. Model Development Process

Accurate yield prediction necessitates a correct understanding of the functional relationship between oil palm yield and the influencing factors. To reveal such a relationship, a powerful machine learning model is required. However, there is no “one-size-fits-all” model that can perform best in every situation. Therefore, an appropriate model selection is of paramount importance [70].

#### 2.5.1. Model Selection

Developing various prediction models to identify the best model is a tedious task while perfect model selection is still not guaranteed. Therefore an automated model selection was performed using Pycaret 2.0 Python library [71] where all existing regression models were trained and compared automatically based on the defined preprocessing pipeline for the given data set. Furthermore, the performance of models was optimized using the hyperparameters tuning. The main purpose of the experiment was to identify the appropriate regression model for oil palm yield prediction.

#### 2.5.2. Model Building

From the list of recommended models, two top models were created and refined: (1) Extra Tree (Extremely Randomized Tree) Regressor and (2) AdaBoost (Adaptive Boosting) Regressor. A brief description of each model is given in the succeeding sections.

- Extra Tree Regressor: Theoretical background and its application in the prediction problem

**Figure 3:**
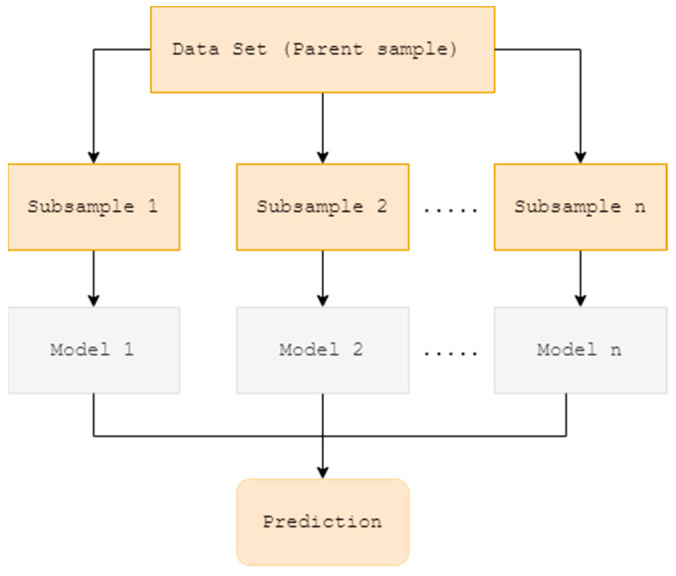
Schematic diagram of the Extra Tree Regressor.

An Extra Tree learns from parent sample by splitting main data into numerous subgroups (child samples) to obtain a prediction from each subgroup individually. It produces final prediction from the combined predictions of all subgroups. Averaging is used to improve the prediction while simultaneously dealing with overfitting [10]. The model separates subgroups by selecting random split points, which makes it different from other tree-based ensembles [11]. Its two primary distinctions from the classical tree-based ensemble methods are: (1) dividing subgroups at random and (2) growing trees using the full learning sample [72]. The schematic diagram of Extra Tree Regressor is provided in the Figure 3.

- AdaBoost Regressor: Theoretical background and its application in prediction problem

**Figure 4:**
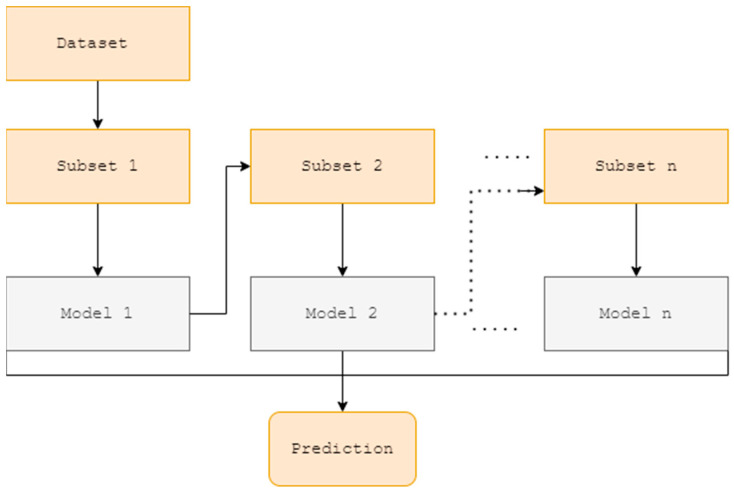
Schematic diagram of the AdaBoost Regressor.

AdaBoost is a statistical classification and regression algorithm that works by sequentially generating multiple regressors to finalize a weighted model [73]. The model can automatically adjust the weights based on estimation errors; therefore, it has great potential for addressing nonlinear, complicated regression problems [74]. AdaBoost develops numerical models by altering the distribution of the parent sample. Once the samples are chosen based on accuracy, all the weak predictions are boosted by the same amount. Therefore, AdaBoost maintains a better performance than other models even in the existence of noise in the data. As a result, it may be less prone to the overfitting problem than other learning algorithms in particular situations. Even though individual learners may be poor, as long as their performance is marginally better than random guessing, the total model will converge to a powerful learner [75]. The schematic diagram of AdaBoost is provided in Figure 4.

#### 2.5.3. Performance Evaluation and Comparison via Evaluation Matrices

The performance evaluation metrics were used to monitor and measure the performance of the models. The two highest performing models were selected from the stack of best to worst performing models based on six evaluation matrices namely mean absolute error (MAE), mean squared error (MSE,) root mean squared error (RMSE), coefficient of determination or R squared error (R^2^), root mean square logarithmic error (RMSLE), and mean absolute percentage error (MAPE), while considering R^2^ as the key performance indicator (KPI). Further description of the aforementioned evaluation matrices can be seen in [2]. In addition, the models’ performance was compared with several other models to confirm the significance and to prove the superiority of the selected models.

## 3. Results

### 3.1. Model-Based Feature Importance

**Figure 5:**
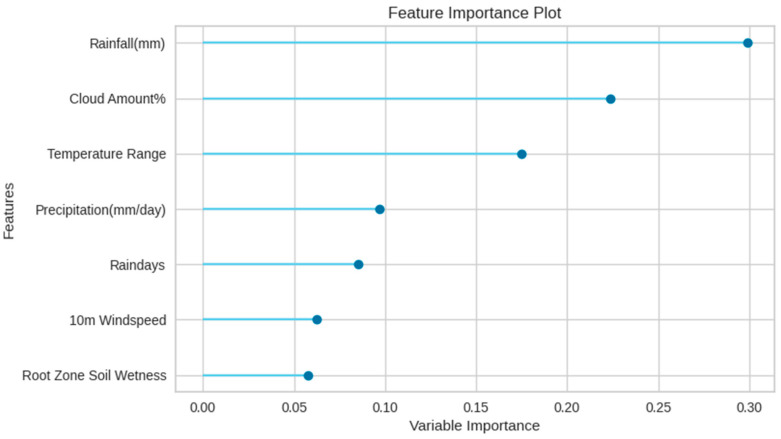
Feature importance plot of Extra Tree.

**Figure 6:**
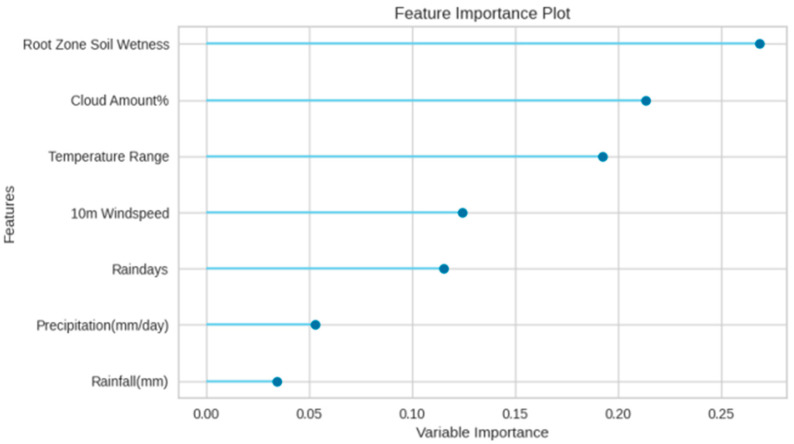
Feature importance plot of AdaBoost.

The feature importance plots simplify the differences between the working mechanism of the models. One of the main distinctions between the algorithms is that the Extra Tree model picks the feature based on its accuracy and assigns high associated value to the strong learners. The AdaBoost Regressor learns from errors and prioritizes features with lower accuracy and assigns higher associated values to weak learners [76]. Meanwhile, the Extra Tree Regressor assigned high weights to the features causing the lowest error and vice versa. Model-based feature importance specifies that the Extra Tree regression algorithm takes root zone soil moisture as the strongest feature and rainfall reflects the least feature value. Cloud amount, temperature range, wind speed, and the number of rain days gave the most to least feature values, respectively. Unlike the Extra Tree model, AdaBoost Regressor assigned the highest features values to the error-prone features. Rainfall gave the highest importance with a feature value of 0.30 and root zone soil moisture was ranked last with the lowest feature value. It indicates that root zone soil wetness feature aided in high error compared to the rest of the features. Caused by the unique feature importance strategy of each algorithm, cloud amount and temperature range were given lower associated values in AdaBoost compared to Extra Tree. Feature importance plots of Extra Tree and AdaBoost are shown in Figure 5 and Figure 6, respectively.

### 3.2. Evaluation of the Extra Tree and AdaBoost Regressors via Residuals, Prediction Error

**Figure 7:**
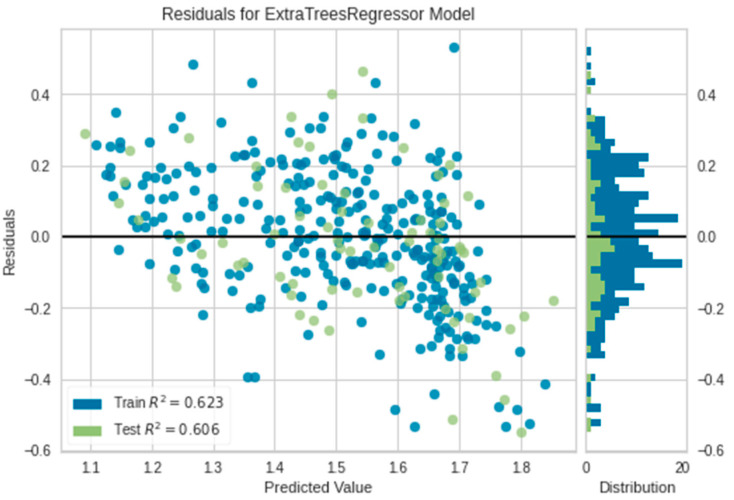
The residual plot of Extra Tree.

**Figure 8:**
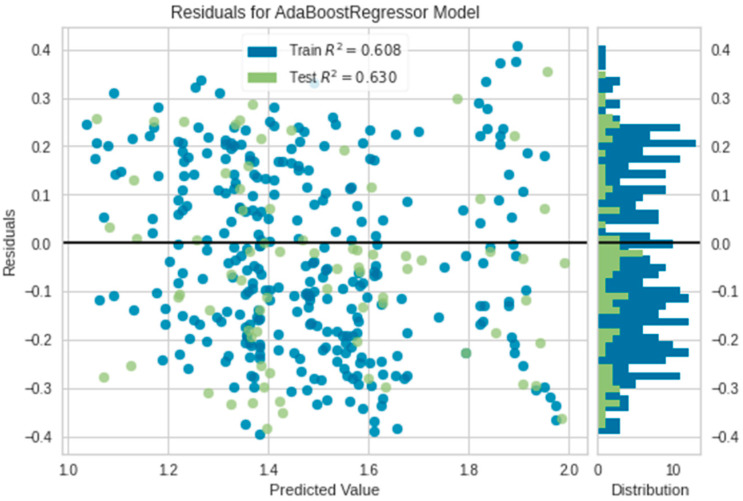
The residual plot of AdaBoost.

From the above evaluation, the tree-based ensemble models, i.e., Extra Tree and AdaBoost Regressor exhibit the ability to predict oil palm yield with mean R^2^ values 0.6057 and 0.63, respectively. The R^2^ scores in Figure 7 and Figure 8 specify the goodness of fit of the underlying regression models to the test data. The residual plots of the models display inconsistent over-predicted and under-predicted values above and below the fitting line, respectively. The residuals of AdaBoost are more scattered compared to the residuals in the Extra Tree residual plot. This indicates the sensitivity of Extra Tree to the data disparity. The Extra Tree Regressor could learn the training and testing data better than the AdaBoost Regressor which slightly overfits.

**Figure 9:**
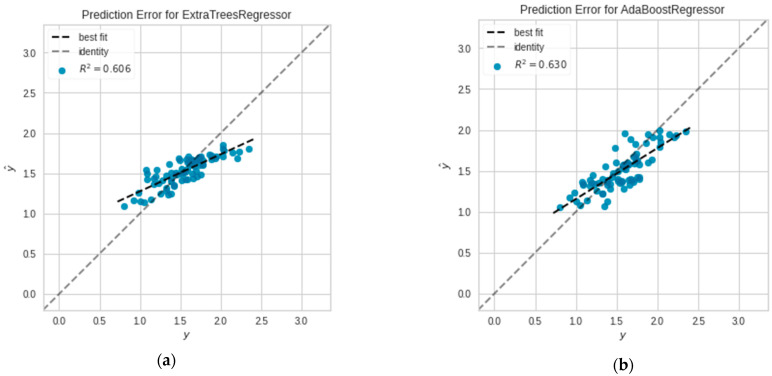
Prediction error of (**a**) Extra Tree; (**b**) AdaBoost.

The actual targets from the dataset were compared to the projected values generated by the study’s models in a prediction error plot. This provides the picture of how much variation the models had. As indicated in the error plots of the models in Figure 9a,b the prediction by Extra Tree and AdaBoost inclined to a specific point where y and ŷ denote the actual values and predicted values, respectively. Nevertheless, the prediction error of Extra Tree tends to increase proportionately to the data points comprising of extreme values. It can be seen in both plots that values are concentrated down to small range of the target feature as the oil palm yield remained 1–2.5 tons/hectare in Pahang throughout the study period.

### 3.3. Evaluation of Extra Tree and AdaBoost via Learning Curve and Validation Curve

**Figure 10:**
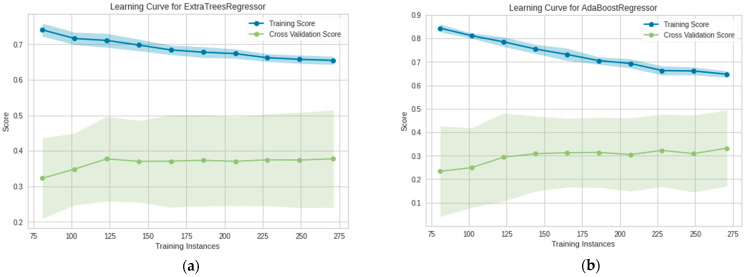
Learning curve of of (**a**) Extra Tree; (**b**) AdaBoost.

The training and testing curves shown in Figure 10a,b were used to evaluate the performance of machine learning models on the training and testing data. As observed, the gap between training and testing scores tends to decrease with the increase of training instances. This indicates: (1) the inadequacy of data size for the models to show optimum performance and (2) current features explaining 60% yield variability that can be seen from R^2^ since crop protection records were not included in the data. Thus, more training instances and new information (features) can be added in order to increase the prediction accuracy. Fortunately, the workflow is also capable of capturing the omitted variable bias, this also reflects the impact of missing features in the data.

**Figure 11:**
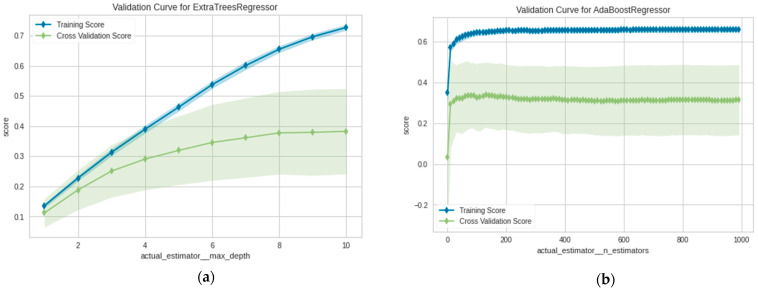
Cross validation of (**a**) Extra Tree; (**b**) AdaBoost.

Accordingly, the validation curves of the Extra Tree and AdaBoost Regressors are presented in Figure 11a,b, respectively. The learning curve generated from a holdout test dataset indicates how the models can effectively generalize the dataset [77]. The Extra Tree Regressor appeared to be more stable and iteratively improved while the AdaBoost Regressor was more underperformed. Besides cross-validation and statistical evaluation metrics, another method to verify model’s performance is by obtaining the predictions on unseen data.

**Figure 12:**
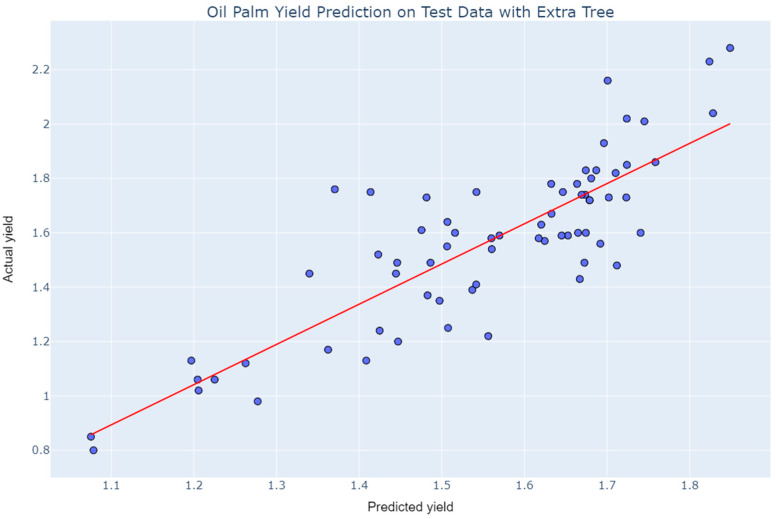
Prediction of oil palm yield by Extra Tree.

**Figure 13:**
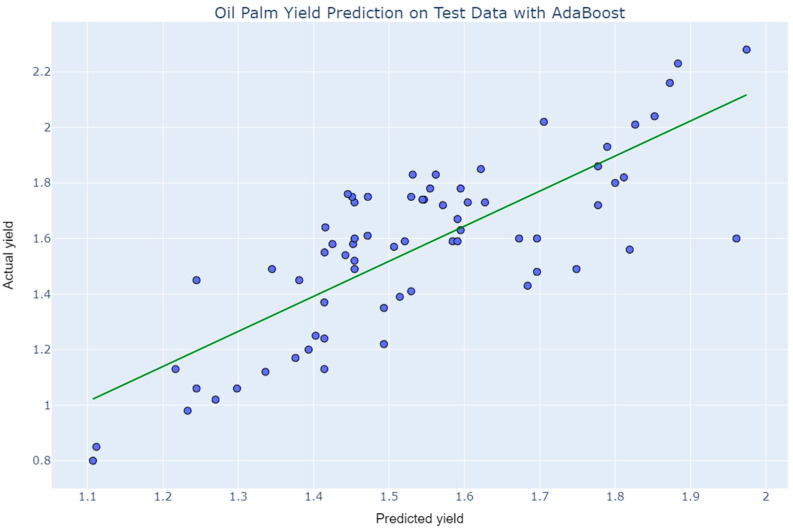
Prediction of oil palm yield by AdaBoost.

The results from cross-validation were verified by predicting unseen data. Extra Tree slightly overperformed and reflected better generalization than the AdaBoost Regressor. The predictions on unseen data of Extra Tree and AdaBoost are presented in Figure 12 and Figure 13, respectively. Although the real-world multisource data is too complex to be predicted accurately, the precisions of the data are better than expected for a small sample size.

### 3.4. Comparative Analysis of Selected Models with Tree-Based Regressors

In this section, the performance of the proposed oil palm yield prediction framework was compared against some latest and most popular conventional tree-based machine learning models under an identical preprocessing pipeline and same feature set. The 10-fold cross-validation technique was employed in conjunction with the performance evaluation metrics to evaluate the performance of the regressors. Tree-based regressors are selected in the proposed framework. Therefore, the comparison analysis was performed with other similar models such as Random Forest, Gradient Boosting Tree, and Decision Tree. The selected models outperformed the conventional tree-based machine learning models in terms of evaluation matrices. The corresponding results are provided in Table 2 where performance superiority of the selected models can be observed from outcomes. Extra Tree achieved low MAE, MSE, RMSE, RMSLE, and MAPE (0.1562, 0.0405, 0.2013, 0.0788, and 0.106, respectively) with coefficient of determination (R^2^) of 0.6057. Likewise, AdaBoot obtained nearly equal value of R^2^ as Extra Tree, which was significantly better than other tree-based models.

### 3.5. Comparative Analysis of Selected Models with Conventional Regression Methods

**Figure 14:**
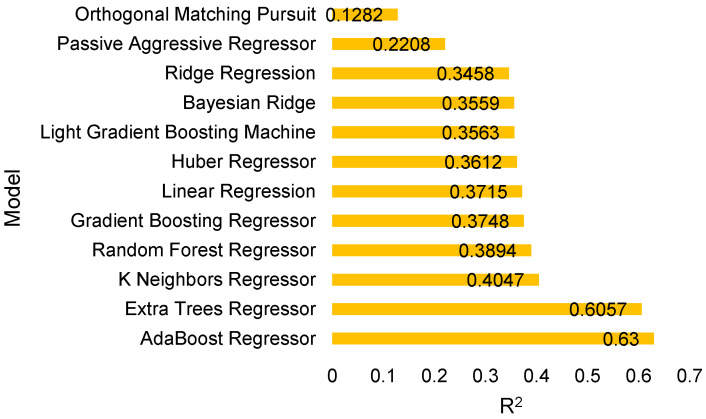
KPI based performance comparison of different models.

In addition, the performance of selected models was compared to other non-tree-based models under an identical process. From the results, it can be observed that the tree-based models outperformed other regression models such as Multiple Linear Regression, Least Angle Regression, Bayesian Ridge Regression, Huber Regressor, K Nearest Neighbors, Orthogonal Matching Pursuit, Elastic Net Regressor, Passive Aggressive Regressor and Least Absolute Shrinkage, and Selection Operator (Lasso) Regressor. Furthermore, the overall performance of all tree-based regression models is considerably better in the oil palm yield prediction. The KPI based performance comparison between the aforementioned models with the selected models in this study is presented in Figure 14.

## 4. Discussion

Weather extremes and variability have a significant impact on agricultural systems. Understanding the impacts of climate on oil palm production is a critical step in assessing its resilience to weather variations and developing appropriate strategic changes [78]. In this study, machine learning regression algorithms in supervised machine learning reflected the strength to learn complex patterns from agro-metrological data. As the results suggest, weather impacts on yield variations were exhibited in nonlinear dependencies among data variables. Besides predicting future yield from historical observations, the machine learning methods could also indicate data redundancy and insufficient input.

### 4.1. Interpretability of the Models

#### 4.1.1. Feature Selection

Since the complicated relationships between weather and crop variations require as much information as possible, the number of available features limits the complexity of the model. An input selection is crucial to reduce the overfitting issue, which is a situation when a machine learning model works well on training data. However, models that overfit are more likely to show poor predictive performance on new data. For instance, overfitting occurs when a model is excessively complex, such as obtaining a high number of features in a small sample size (insufficient data) to regulate it. The feature selection methods not only identify the inputs that are more correlated to oil palm yield, they also ensure that only the most complementary features are selected. However, it is not clear as to what extent the selected features could reduce or increase the yield values.

Regarding data utilization, much consideration is required when using multisource data with different temporal resolutions and multiple feature measuring units in order to avoid false conclusions and non-reliable results. To solve this issue, unit conversion, feature scaling, and data split into independent years were used in the analysis for the learning and validation sets. To define a learning set, 70% of data were randomly chosen for training and the remaining 30% were used as the test set. Some other pre-processing steps like commonly adopted data cleaning techniques, such as outlier removal, were applied to improve data quality for prediction.

#### 4.1.2. Interpretation of the Models

Recently, there has been a huge yearning toward the interpretation of tree-based regressors for developing yield prediction models [79,80]. Overall, this study showed that the tree-based ensemble models lead to more better results compared to other baseline machine learning models, such as random forest. In a situation where a linear relationship between the dependent and independent variables exists, a multiple linear models may outperform the tree-based models. When there are great nonlinearity and complex relationship between the dependent and independent variables, a tree-based ensemble model outperforms a typical regression strategy and linear regression.

Generally, the tree-based models are considered easier to comprehend than the linear regression models. Meanwhile, the ensemble models over-perform conventional tree-based models, besides providing a reasonable explanation of features that are important in determining the crop yield values. However, it is not clear to what extent these features are reducing or increasing the yield values. Moreover, it is necessary to maintain a trade-off between the prediction accuracy and interpretability of the selected models. It is also confirmed that the coupling of multiple exploratory methods increases the explainability of the models.

#### 4.1.3. Reusability of the Workflow

The proposed workflow is initially designed for the prediction of oil palm yield in the state of Pahang, Malaysia. However, the results suggest that the flexible workflow could ensure its reusability beyond other traditional crop-specific and site-specific models. The same workflow can be utilized to predict the yield of other crops with different data set from diverse area. In order to do so, it is essential to modify the data preprocessing pipeline accordingly. For instance, processes such as handling missing values, dealing with categorical features (if any), and over sampling/under-sampling of the data (if needed) should be decided carefully in the context of input data. Similarly, the selected models and features may vary as per input and crop responses. Several regression models can be selected, trained, compared, and evaluated following the proposed framework. So far, this workflow offered a set of suitable ensembles, tree-based and other regression models with high precision for oil palm yield prediction. The proposed models may be further refined in future if the work could benefit from more explicit data from soil analysis, disease assessments, fertilizers applications, and irrigation records. In addition, multiple machine learning ensembles can be combined using the stacking methods to improve prediction accuracy.

#### 4.1.4. Limitations of the Workflow

The workflow was initially proposed to check feasibility of machine learning application for oil palm yield prediction. However, there were some constraints and limitations of this work. First, although oil palm yield is determined by several biotic and abiotic factors including management practices and crop protection, so far it is not possible to quantify the impacts of biotic factors such as weeds, disease, pests, and insects on crop performance [81]. Therefore, yield records have to be considered in an ideally protected environment with perfect field management. Secondly, the current workflow followed standard data preparing techniques used in machine learning. As a result, the workflow considered normal weather conditions, while rare extreme events such as heavy raining, flooding, and drought were not recognized. Drought is an important abiotic stressor that can reduce 15% yield on good soil and up to 20% yield on poor soil against 100-mm water deficit [82]. Along with irrigation records, drought should be included in the data to explain water-limited yield variability. Next, this quantitative study also did not examine the impacts of seeds quality, physiochemical properties of the soil, and environmental pollution on oil palm productivity.

Furthermore, data within range of 1986 to 2020 were used to for training, testing, prediction and validation. Making predictions beyond the study period would still require parallel weather data. Weather data can be forecasted using vector auto regressor for multivariate prediction [83,84]. In addition, the method as explained in [85] can also be followed for weather predictions prior to yield predictions.

## 5. Conclusions

This paper presents a novel flexible workflow for oil palm yield prediction using supervised machine learning integrated with (auto-ML) methods. The proposed workflow demonstrated promising performance to accurately predict oil palm yield with the help of several weather-related and soil moisture-based parameters. Furthermore, the auto-ML technique provided solutions for best model selection by automatically selecting best-suited models determined by the pre-processing pipeline. Likewise, the optimum feature subset was selected using the Boruta algorithm that played a key role in data reduction to improve prediction accuracy. In addition, the automated model selection specified two tree-based algorithms, namely Extra Tree Regressor and AdaBoost Regressor which had higher R^2^ value than other existing models. Following this, the selected models were trained and tuned to achieve a realistic precision along with reasonable explanation ability. The performance of the models was evaluated statistically through six different evaluation metrics. The identical evaluation metrics were used to perform a comparison with other state-of the-art similar (tree-based) and dissimilar (non-tree) models. Researchers would be able to execute repeatable experiments for multiple sites of oil palm plantations with standard input data and to achieve repeatable outcomes through replicable techniques.

The use of new data sources and more advanced algorithms as well as extraction of new features from trends and interactions of given features are some of the improvements that could be used to refine the model for certain species of oil palm planted in different locations. Additionally, the reusability of the proposed flexible workflow enables yield prediction of other crops with different data sets containing crop-specific parameters and site-specific historical meteorological observations. On top of that, the capability of the machine learning models in learning complex patterns from multisource meteorological and agricultural data has provided a great potential for their applications towards ensuring sustainability oil palm as an integral part of precision agriculture.

## Figures and Tables

**Table 1:** A detailed summary of input data for yield modeling.

Category	Variable	Spatial Resolution	Temporal Resolution	Time Coverage	Source
Crop data	Yield (t/h)	NA	1 Month	1986–2020	MPOB
Soil moisture data	Surface soil wetness (%)	10 m	1 Month	1986–2020	NASA
Soil moisture data	Profile soil wetness (%)	10 m	1 Month	1986–2020	NASA
Soil moisture data	Root zone soil wetness (%)	10 m	1 Month	1986–2020	NASA
Meteorological data	Cloud amount (%)	NA	1 Month	1986–2020	NASA
Meteorological data	Rain days/month	NA	1 Month	1986–2020	MET
Meteorological data	Wind speed (m/s)	10 m	1 Month	1986–2020	NASA
Meteorological data	Rainfall (mm)	10 m	1 Month	1986–2020	MET
Meteorological data	Radiative flux (kW/h)	2 m	1 Month	1986–2020	NASA/MET
Meteorological data	Min temp (°C)	2 m	1 Month	1986–2020	NASA/MET
Meteorological data	Max temp (°C)	2 m	1 Month	1986–2020	NASA/MET
Meteorological data	Earth skin temp (°C)	2 m	1 Month	1986–2020	NASA/MET
Meteorological data	Temperature range (°C)	2 m	1 Month	1986–2020	NASA/MET
Meteorological data	Surface pressure (kpa)	2 m	1 Month	1986–2020	NASA/MET
Meteorological data	Relative humidity (%)	2 m	1 Month	1986–2020	NASA/MET
Meteorological data	Specific humidity (%)	2 m	1 Month	1986–2020	NASA/MET
Meteorological data	Precipitation (mm)	2 m	1 Month	1986–2020	NASA/MET

**Table 2:** Performance comparison of tree-based models.

Model	MAE	MSE	RMSE	R^2^	RMSLE	MAPE
Extra Tree	0.1562	0.0405	0.2013	0.6057	0.0788	0.106
AdaBoost	0.1602	0.038	0.1951	0.63	0.0779	0.1073
Random Forest	0.1815	0.0534	0.2279	0.3894	0.0922	0.1289
Decision Tree	0.2505	0.1018	0.3161	−0.2015	0.1273	0.1750
Gradient Boosting	0.1836	0.0545	0.2309	0.3748	0.0931	0.1301

## Data Availability

The data that support the findings of this study are openly available at https://power.larc.nasa.gov/data-access-viewer/ (accessed on 4 June 2021), and https://www.mpob.gov.my/ (accessed on 23 September 2021).
